# Nomogram for Predicting Postoperative Portal Venous Systemic Thrombosis in Patients with Cirrhosis Undergoing Splenectomy and Esophagogastric Devascularization

**DOI:** 10.1155/2022/8084431

**Published:** 2022-11-04

**Authors:** Miao Chen, Jian-Bo Han, Jia-Kang Zhang, Qing-Hua Shu, Yu-Feng Zhang, Yong-Xiang Yi

**Affiliations:** Department of Surgery, The Second Hospital of Nanjing, The Affiliated Hospital of Nanjing University of Chines Medicine, Nanjing 210003, Jiangsu, China

## Abstract

**Objectives:**

The aim of the study is to develop a nomogram for predicting postoperative portal venous systemic thrombosis (PVST) in patients with cirrhosis undergoing splenectomy and esophagogastric devascularization.

**Methods:**

In total, 195 eligible patients were included. Demographic characteristics were collected, and the results of perioperative routine laboratory investigations and ultrasound examinations were also recorded. Blood cell morphological traits, including the red cell volume distribution width (RDW), mean platelet volume, and platelet distribution width, were identified. Univariate and multivariate logistic regressions were implemented for risk factor filtration, and an integrated nomogram was generated and then validated using the bootstrap method.

**Results:**

A color Doppler abdominal ultrasound examination on a postoperative day (POD) 7 (38.97%) revealed that 76 patients had PVST. The results of the multivariate logistic regression suggested that a higher RDW on POD3 (RDW3) (odds ratio (OR): 1.188, 95% confidence interval (CI): 1.073–1.326), wider portal vein diameter (OR: 1.387, 95% CI: 1.203–1.642), history of variceal hemorrhage (OR: 3.407, 95% CI: 1.670–7.220), and longer spleen length (OR: 1.015, 95% CI: 1.001–1.029) were independent risk parameters for postoperative PVST. Moreover, the nomogram integrating these four parameters exhibited considerable capability in PVST forecasting. The nomogram's receiver operating characteristic curve reached 0.83 and achieved a sensitivity and specificity of 0.711 and 0.848, respectively, at its cutoff. The nomogram's calibration curve demonstrated that it was well calibrated.

**Conclusion:**

The nomogram exhibited excellent performance in PVST prediction and might assist surgeons in identifying vulnerable patients and administering timely prophylaxis.

## 1. Introduction

Liver cirrhosis has become the 11^th^ most lethal disease worldwide, causing approximately 100 million deaths every year [1]. Portal hypertension (PH) is a typical manifestation among patients with cirrhosis and is mainly characterized clinically by hypersplenism and portosystemic collateral varices. Notably, the rupture of esophagogastric varices (EV) is a common complication that can be lethal without timely hemostasis. Certain differences exist in the management of variceal hemorrhage between Western and Asian doctors. Medication, endoscopic ligation, and intervention therapy are first-line treatments in Western countries [2]. However, the etiology of cirrhosis in China and Western countries is different: liver cirrhosis in China is mainly derived from hepatitis B virus infection, characterized by poor liver function and affecting a large population, whereas alcoholic and nonalcoholic hepatitis and hepatitis C virus infection account for most cases of cirrhosis in Western countries [3]. Splenectomy combined with esophagogastric devascularization (SED) is widely performed in China because of its great ability to decrease pressure in portal veins, ameliorating the degree of EV, and improving immune function without harming liver function [4]. For patients admitted with emergent uncontrollable bleeding, SED is quite effective in hemorrhage control and has a lower rebleeding rate than endoscopic therapy and higher economic benefits than other therapies in the long run [5]. Thus, SED is still an important treatment for PH in China.

However, portal venous systemic thrombosis (PVST) (defined as the formation of a thrombus in either the intrahepatic portal vein, extrahepatic portal vein, splenic vein, or superior mesenteric vein) is a common and serious complication following SED, with an incidence rate ranging from 18.9% to 55% [6, 7]. Furthermore, the incidence rate of spontaneous PVST in the general population with cirrhosis is 5%–15% [8], indicating a prevalence of thrombophilia among patients receiving SED. In patients with PH, PVST has been associated with a high rebleeding rate and liver transplantation failure [9,10]. Although PVST may manifest as abdominal discomfort or continuous low-grade fever, it is mostly asymptomatic until complications occur, making timely prophylaxis challenging. Thus, solving how to assess the risk of early PVST after SED is crucial for the prognosis of patients with cirrhosis.

To date, studies investigating PVST following SED have reported various risk factors, including a wider portal vein diameter (PVD), poorer liver function, prolonged prothrombin time (PT), higher levels of preoperative aspartate aminotransferase (AST) and alanine aminotransferase (ALT), and postoperative thrombocytosis [11–14]. However, a consensus has yet to be achieved. A nomogram is an effective tool for the visualization and application of regression models and can be helpful for risk assessment and clinical decision-making [15]. In this study, we developed a nomogram for the risk evaluation of postoperative PVST by retrospectively analyzing the hospitalization data of patients with cirrhosis undergoing SED to provide evidence for the early prophylaxis of PVST.

## 2. Methods

### 2.1. Study Design and Participants

This single-center retrospective case-control study conformed to the Declaration of Helsinki (as revised in 2013) and was approved by the Medical Ethics Committees of the Second Hospital of Nanjing.

Patients with cirrhosis who underwent open SED at the Department of Hepatobiliary Surgery, Second Hospital of Nanjing, between January 2013 and December 2018 were included. A detailed description of the SED procedure is provided in the following section. The inclusion criteria were as follows: (1) patients aged between 18 and 75 years, (2) patients diagnosed with liver cirrhosis based on pathological or radiological evidence, (3) patients with PH and severe EV (varices in the form of a serpent, nodule, or tumor or moniliform with or without red signs), (4) patients with a platelet count <50 × 10^9^/L, white blood cell (WBC) count <3 × 10^9^/L, and/or with a history of variceal bleeding resulting from PH, and (5) patients with a Child-Pugh score of A or B.

The exclusion criteria were as follows: (1) patients with hepatocellular carcinoma or other malignant diseases identified before or during surgery, (2) patients with PVST prior to surgery, (3) patients with hematological or immune system diseases, (4) patients who had received anticoagulants prior to surgery, (5) patients with organ failure or refractory ascites, and (6) patients with missing data.

### 2.2. Data Collection

Basic data including age, sex, body mass index (BMI), history of hypertension, history of diabetes mellitus, history of variceal hemorrhage, cirrhosis etiology, the presence of ascites, emergency at admission, the model for end-stage liver disease (MELD) score, Child-Pugh score, PVD, spleen thickness, spleen length, and portal venous flow velocity were recorded. Preoperative laboratory blood tests to measure WBC, red blood cells (RBCs), RDW, platelet distribution width (PDW), mean platelet volume (MPV), hematocrit, coagulation parameters, AST, ALT, total bilirubin, albumin, globin, and postoperative RDW, PDW, MPV, and hematocrit were also collected on a postoperative day (POD) 1, 3, and 7.

All the patients received an abdominal color Doppler ultrasound examination on POD7, and the diagnosis of PVST was confirmed by two experienced imaging experts. Ultrasound follow-up was conducted every 2 weeks for all patients.

### 2.3. Surgical Procedure

All patients underwent SED using laparotomy. The abdomen was opened using an L-shaped left subcostal incision, and a splenectomy was performed. The peripheral ligaments of the spleen, including the gastrocolic ligament, splenocolic ligament, gastrosplenic ligament, splenorenal ligament, and splenophrenic ligament, were cut and suture ligated. The splenic hilum was rigorously dissected, and the splenic artery and vein were carefully transected and ligated. Soon after splenectomy, devascularization was conducted. The portosystemic collateral branches at the lower esophagus and fundus of the stomach, including the short gastric vein, posterior gastric vein, left inferior phrenic vein, and esophagogastric branches of the gastric coronary vein, were identified and ligated. The esophagus was dragged down, and the high esophageal branches of the left gastric vein were sutured at a distance of approximately 10 cm from the fundus of the stomach; the arteries accompanying these veins were divided accordingly.

### 2.4. Statistical Analysis

The normality of continuous data were analyzed using the Shapiro–Wilk test. The Student's *t*-test was performed on data with normal distribution, and the Mann–Whitney *U* test was used for nonparametric tests. Categorical variables were compared using either the Chi-square test or Fisher's exact test. Normally distributed continuous variables are presented as the mean (standard deviation) and nonnormal variables as the median (interquartile range, IQR). Categorical variables are presented as the exact number (percentage, %). Significant variables in the univariate comparison were further analyzed using a multivariable stepwise logistic regression.

The nomogram was developed based on the multivariate logistic regression model using the “rms” R package. The bootstrap method [16] (1,000 resamples) was used for internal validation, and the corresponding calibration and receiver operating characteristic (ROC) curves were plotted using “plotROC” R packages. The cutoff value of the ROC was calculated according to the Youden index [17]. A two-tailed *p* value of less than 0.05 was considered significant throughout the analysis. R software (version 3.6.1) was used for all the statistical analyses.

## 3. Results

### 3.1. Participant Characteristics

**Figure 1:**
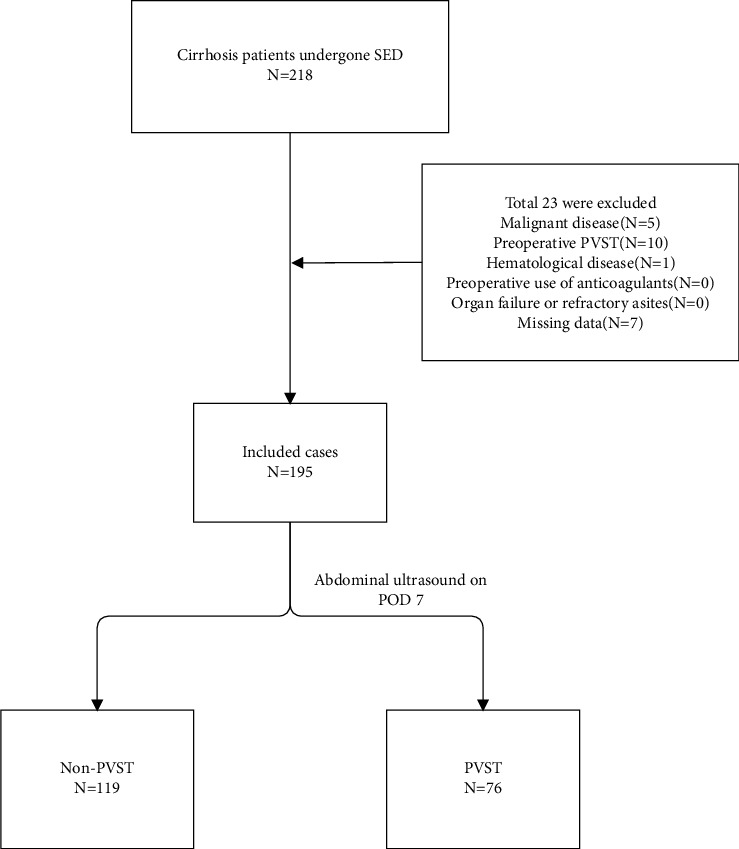
Study flowchart.

A total of 218 patients with cirrhosis were admitted and underwent SED for either the primary or secondary prevention of variceal hemorrhage at the Department of Hepatobiliary Surgery, Second Hospital of Nanjing, between January 2013 and December 2018. A total of 23 patients were excluded, in line with the exclusion criteria, as follows: 10 with preoperative PVST, 5 with hepatocellular carcinoma, 1 with thalassemia, and 7 without the required data. The study flowchart is presented in Figure 1. Therefore, 195 eligible patients were included in the analysis. The abdominal color Doppler ultrasound examination on POD7 (38.97%) revealed that 76 of these patients had developed PVST and they were assigned to the study group; the remaining 119 patients without PVST were assigned to the control group. The location of the PVST is illustrated in Supplementary Table 1.

The age of the entire cohort ranged from 23 to 72 years, with an average age of 47.8 years. Men constituted 128 (65.64%) of the patients. The commonest cause of cirrhosis was infection with hepatitis B (140 patients), with the other causes being hepatitis C infection (34 patients), alcoholic hepatitis (14 patients), primary biliary cirrhosis (4 patients), drug-induced cirrhosis (2 patients), and schistosomiasis cirrhosis (1 patient).

All patients underwent SED using laparotomy (the surgical procedure is described above). Detailed data from the PVST and non-PVST subgroups are presented in Table 1. There was no statistical difference in age, sex, cirrhosis etiology, history of diabetes, hypertension, or BMI between the two groups.

### 3.2. Factor Comparison between the Groups

No significant differences were identified in the velocity of portal blood flow, spleen thickness, emergency operation rate, Child-Pugh score, MELD score, or ascites between the PVST and non-PVST groups. Regarding the preoperative laboratory indicators, no significant differences were detected in RBC count, neutrophil count, monocyte count, platelet count, MPV, PDW, PT, activated partial thromboplastin time, thrombin time, total bilirubin, ALT, albumin, or globin between the two groups. However, variceal bleeding was more common in the PVST group (59.2% vs. 38.7%, *p*=0.008). In the preoperative ultrasound imaging, the spleen length was greater in the PVST group than in the non-PVST group (186 vs. 170 mm, *p* < 0.001), and the PVD was wider in the PVST group (14.75 vs. 12.80 mm, *p* < 0.001). The PVST group had significantly lower hemoglobin levels at admission (93.97 vs. 101.97 g/L, *p*=0.012). RDW was significantly higher in the PVST group either before surgery (17.15% vs. 15.6%, *p*=0.002) or on POD1 (16.9% vs. 15.4%, *p*=0.004), POD3 (18.65% vs. 15.4%, *p*=0.001), or POD7 (16.00% vs. 15.60%, *p*=0.044). In comparison with the non-PVST group, the PVST group had lower values in the following parameters: lymphocyte count (0.48 vs. 0.59 10^3^/uL, *p*=0.001), hematocrit (27.95% vs. 29.90%, *p*=0.013), fibrinogen (1.39 vs. 1.67 g/L, *p*=0.003), and AST (24.1 vs. 29.6 U/L, *p*=0.011).

### 3.3. Independent Risk Factor Analysis Using Multivariate Logistic Regression

To screen for independent parameters associated with the development of PVST, factors identified as significant in the univariate comparison were included in the multivariate logistic regression: PVD, history of bleeding, spleen length, WBC, RDW, hematocrit, lymphocyte, hemoglobin, AST, and fibrinogen. Following the stepwise elimination (direction = both) of the nonsignificant variables, PVD (OR: 1.387; 95% CI: 1.203–1.642), spleen length (OR: 1.015; 95% CI: 1.001–1.029), bleeding history (OR: 3.407; 95% CI: 1.670–7.220), and RDW on POD3 (RDW3) (OR: 1.188; 95% CI: 1.073–1.326) were identified as independent risk factors for PVST. The results of the multivariate regression are displayed in Table 2. WBC, hematocrit, lymphocyte, hemoglobin, AST, and fibrinogen were eliminated from the final model.

### 3.4. Performance of the Risk Factors and Nomogram

**Figure 2:**
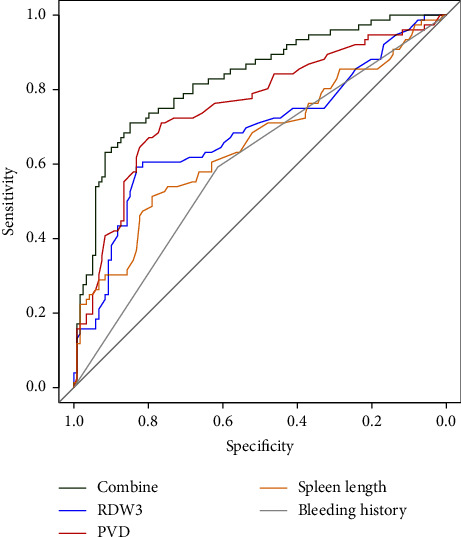
Receiver operating characteristic analysis of the independent risk factors. PVD: portal vein diameter, RDW3: red blood cell distribution width on postoperative day 3, and Combine: PVD + RDW3 + history of bleeding + spleen length.

A ROC analysis was conducted to validate the performance of the independent risk factors and logistic model in forecasting postoperative PVST. The ROC curves of the individual risk parameters are presented in Figure 2. The area under the curve (AUC) and the sensitivity and specificity at their respective cutoffs are listed in Table 3. The AUC for the RDW3, PVD, spleen length, and history of bleeding were 0.685 0.757, 0.655, and 0.60, respectively. The model integrating the four parameters achieved an AUC of 0.83, with a sensitivity and specificity of 0.711 and 0.849, respectively, indicating the superiority of the model over any individual factor in PVST forecasting.

**Figure 3:**
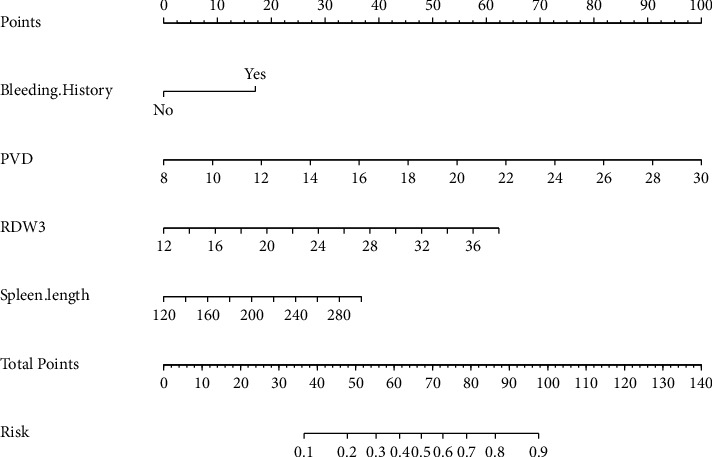
Nomogram based on portal vein diameter, history of bleeding, spleen length, and red blood cell distribution width on postoperative day 3.

**Figure 4:**
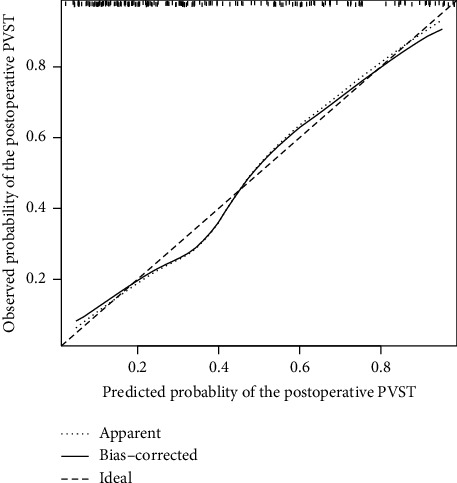
Calibration curve of the nomogram for the prediction of portal venous systemic thrombosis (PVST). The *x*-axis represents the predicted risk of PVST using the nomogram, and the *y*-axis represents the observed risk.

The nomogram was generated using a logistic model (Figure 3). Each risk factor value in the nomogram was assigned a weighted score from the point bar at the top, and their sum was mapped to the risk bar at the bottom, representing the PVST risk of an individual. The bootstrapped concordance index (C-index) of the nomogram was 0.83, emphasizing its excellent discrimination capability. A calibration curve was plotted for the internal validation of the nomogram (Figure 4), which indicated that the predicted risk was in close agreement with the observed scenario.

## 4. Discussion

PH and hypersplenism are common complications of cirrhosis. Splenectomy greatly reduces pressure in the portal system and ameliorates variceal bleeding and ascites in patients with cirrhosis; however, it also increases patients' exposure to infection [18–20]. Splenic arterial embolism, which has the advantages of less trauma, a quicker recovery time after surgery, and a confirmed curative effect on hypersplenism, is recognized as a surrogate modality for splenectomy [21]. Unfortunately, both splenectomy and splenic arterial embolism have an elevated risk of postoperative PVST [7, 22], with a recent meta-analysis demonstrating that the incidence of postoperative PVST after these two surgical procedures were statistically similar [23].

One study reported that patients undergoing SED had a relatively high incidence of postoperative PVST (18.9%–55% vs. 5%–15%) [6]. The incidence of postoperative PVST following SED in this study was 38.97% (76/195), which is consistent with the reported incidence.

To date, multiple factors, such as postoperative thrombocytosis, a wider preoperative splenic vein diameter, faster portal blood flow, prolonged PT, larger spleen volume, and devascularization, have been reported as risk factors for postoperative PVST [11, 24] but a consensus is yet to be reached. Our univariate and multivariate analyses identified RDW3, PVD, spleen length, and a history of bleeding as independent risk factors for PVST after SED. The nomogram integrating these four factors exhibited an excellent performance in PVST prediction, achieving an AUROC of 0.83.

This study identified that preoperative PVD was an independent risk factor for postoperative PVST (OR: 1.387; 95% CI: 1.203–1.642). Previous studies [14, 25] have demonstrated that a wider preoperative PVD is associated with a greater decrease in blood flow and velocity following spleen resection, resulting in longer blood retention in the portal vein. In addition, the stumps of the dissected vessels were more likely to trigger turbulence and lead to the development of PVST.

This study also revealed spleen length as a risk factor for PVST (OR: 1.015; 95% CI: 1.001–1.029). Spleen volume reflects the severity of PH and the degree of hypersplenism; the larger the spleen is, the more serious the PH, and thus, the greater the likelihood of PVST [26]. In addition, our study demonstrated that patients with cirrhosis with a history of variceal hemorrhage are more prone to PVST (OR: 3.407; 95% CI: 1.670–7.220; *p* < 0.001). Variceal hemorrhage is the manifestation of a decompensated liver, and patients with a history of bleeding have a relatively poor prognosis [27]. Previous studies [28] have proposed that the hypercoagulability of blood may be more severe in patients with cirrhosis with poorer liver function because of their resistance to thrombomodulin activity, a critical factor mediating the anticoagulation process. Consistent with the findings of this study, Xu et al. [29] demonstrated that variceal bleeding was more common in the PVST group than in the non-PVST group (*p*=0.006) but they failed to include this factor in their final model.

The RDW is one of the parameters in routine blood tests—it is an indicator of the variability in erythrocyte size and is frequently overlooked in thrombosis events. Recently, the role of the RDW in thrombotic diseases has attracted significant attention from researchers. Relevant studies have demonstrated that the RDW is a powerful indicator of myocardial infarction, cerebral thrombosis, pulmonary embolism, and atrial fibrillation [30–33]. Lappegard et al. conducted a prospective study of the general population and discovered that individuals with a higher RDW were more likely to experience an incident stroke during follow-up (hazard ratio: 1.55, 95% CI: 1.16–2.06) [34]. Lippi et al. revealed that the RDW (cutoff 14.6%) is an independent risk factor for deep vein thrombosis and pulmonary embolism (OR: 2.52; 95% CI: 1.42–4.47), although the underlying mechanisms have yet to be determined [35].

Increasing evidence suggests that the RDW is a feasible surrogate indicator for systemic inflammatory response and oxidative stress [36–38]. Relevant studies have associated the RDW with typical inflammatory indicators, including the C-reactive protein, erythrocyte sedimentation rate, platelet count, and cytokines such as interleukin (IL)-8 and tumor necrosis factor (TNF) [26, 33]. Patients with cirrhosis often present with chronic inflammation and high coagulability, and the association between the RDW and inflammation may be because the elevation of inflammation factors, such as TNF and IL, aggravates microcirculation hypoxia, resulting in hemodynamic changes. All these changes could suppress the maturity of erythrocytes, cause RBC heterogeneity elevation, and influence the RDW. Thus, the elevated RDW may be attributed to the inflammation response in the body. However, the interactions between inflammation and coagulation are complex. For one thing, the cytokines that trigger the inflammatory cascade can also initiate the thrombosis process and inhibit the physiological mechanisms of anticoagulation. For another, inflammation activity is modulated by the components of coagulation as feedback [39].

This study identified that patients with postoperative PVST exhibited a higher RDW either before or after SED; however, only the RDW on POD3 was an independent risk factor for postoperative PVST (OR: 1.188; 95% CI: 1.073–1.326; *p*=0.001). This may be because systemic inflammation is at its most severe on POD3 and is indicative of a high likelihood of PVST. An excessive inflammatory response may lead to severe epithelial damage, facilitating the adhesion of platelets. Furthermore, considering the higher postoperative RBC levels in the PVST group, it is hypothesized that the elevated RDW reflects the hemopoiesis attributed to the cytokines secreted during inflammation, and this may subsequently result in an increase in blood viscosity.

The current consensus on the prophylaxis of postoperative PVST is to administer anticoagulants once the risk of bleeding is eliminated. Following splenectomy, the prophylactic use of anticoagulants, such as low-molecular-weight heparin and rivaroxaban, can help to reduce the occurrence of PVST [40]. In our center, all the patients who had undergone SED were prescribed low-molecular-weight heparin (4,000 IU, i. h, once a day) for a week as the routine prophylaxis for PVST. Patients diagnosed with PVST using abdominal color Doppler ultrasound on POD7 were prescribed 100 mg of aspirin daily until the portal vein was recanalized. However, the rational selection of patients eligible for anticoagulant therapy is challenging because nonspecific anticoagulation may cause coagulation dysfunction. Thus, we developed a nomogram to identify patients at high risk of postoperative PVST.

This study has some unavoidable limitations. First, because the study had a retrospective design and was conducted in a single center with a small sample size of only 195 patients, a selection bias may be inevitable. Second, the validation of the logistics model was based solely on interval bootstrapping methods. External large-scale sample validation and future multicenter prospective studies are warranted to test the broader application of the proposed model. Third, most of the candidate factors included in the multivariable analysis were selected from the literature, and the independent risk factors may be affected by the inclusion of additional candidates. Last, the value of the RDW is influenced by multiple factors not considered in this study, such as the absorbance of vitamin B12 and iron.

In summary, we proposed a nomogram integrating preoperative PVD, a history of variceal hemorrhage, spleen length, and the RDW on POD3, which exhibited an excellent capability for identifying patients vulnerable to PVST soon after SED. Our nomogram may aid surgeons in forecasting postoperative PVST and administering timely prophylactic treatment to patients at high risk of thrombosis. In the future, appropriate large-scale external validation may confirm the utility of the proposed nomogram in PVST forecasting.

## Figures and Tables

**Table 1:** Characteristics of patients in the non-PVST and PVST groups.

Factors		Non-PVST(119)	PVST(76)	*P* value
*Preoperative*				
Age (years)		48.90 (10.32)	46.16 (9.26)	0.061
BMI (kg/m^2^)		22.60 (20.85, 24.40)	22.80 (20.90, 24.52)	0.81
*Gender (%)*	Female	44 (37.0)	23 (30.3)	0.419
	Male	75 (63.0)	53 (69.7)	
*Diabetes (%)*	No	107 (89.9)	70 (92.1)	0.794
	Yes	12 (10.1)	6 (7.9)	
*Hypertension (%)*	NO	114 (95.8)	76 (100.0)	0.178
	Yes	5 (4.2)	0 (0.0)	
*Emergency (%)*	No	108 (90.8)	73 (96.1)	0.266
	Yes	11 (9.2)	3 (3.9)	
*Child-Pugh (%)*	A	84 (70.6)	58 (76.3)	0.477
	B	35 (29.4)	18 (23.7)	
*Ascites (%)*	No	99 (83.2)	59 (77.6)	0.436
	Yes	20 (16.8)	17 (22.4)	
*Bleeding history (%)*	No	73 (61.3)	31 (40.8)	0.008
	Yes	46 (38.7)	45 (59.2)	
*Cause of cirrhosis (%)*	Others	29 (24.4)	26 (34.2)	0.185
	HBV	90 (75.6)	50 (65.8)	
The thickness of spleen (mm)		56.00(50.50, 63.50)	57.30 (53.00, 67.00)	0.099
Length of spleen (mm)		170.00(155.00, 183.50)	186.00 (165.00, 203.75)	<0.001
PVD (mm)		12.80 (12.00, 13.50)	14.75 (13.20, 16.85)	<0.001
PVV (mm/s)		17.20 (14.95, 20.10)	18.40 (14.78, 20.19)	0.27
MELD		10.00 (9.00, 12.00)	10.00 (9.00, 13.00)	0.526
WBC (10^3^/uL)		2.18 (1.79, 2.90)	1.94 (1.52, 2.66)	0.023
RBC (10^6^/uL)		3.42 (0.63)	3.48 (0.58)	0.537
HB (g/L)		101.97 (21.88)	93.97 (20.91)	0.012
Lymphocyte (10^3^/uL)		0.59 (0.46, 0.82)	0.48 (0.36, 0.64)	0.001
PLT (10^3^/uL)		38.00 (26.50, 52.50)	36.50 (28.00, 53.00)	0.713
Neutrocyte (10^3^/uL)		1.30 (0.98, 1.83)	1.13 (0.86, 1.79)	0.176
Monocyte (10^3^/uL)		0.20 (0.15, 0.28)	0.17 (0.14, 0.29)	0.274
PT (s)		15.30 (13.80, 16.95)	15.55 (14.57, 16.62)	0.625
APTT (s)		40.20 (34.15, 46.10)	40.50 (35.03, 45.42)	0.811
FIB (g/L)		1.67 (1.33, 2.08)	1.39 (1.20, 1.74)	0.003
TT (s)		18.30 (13.20, 21.20)	19.50 (17.08, 21.08)	0.087
HCT (%)		29.90 (26.45, 33.90)	27.95 (25.70, 31.15)	0.013
MPV (fl)		10.40 (9.55, 12.15)	10.30 (9.70, 11.62)	0.718
PDW (fl)		17.30 (15.95, 17.86)	17.16 (16.05, 18.25)	0.608
RDW (%)		15.60 (14.50, 17.35)	17.15 (14.90, 20.30)	0.002
Albumin(g/L)		36.40 (33.40, 39.35)	37.05 (34.18, 40.40)	0.262
Globin (g/L)		27.20 (22.65, 30.30)	26.45 (22.45, 28.77)	0.147
ALT (U/L)		24.70 (17.25, 32.95)	20.25 (16.05, 27.70)	0.093
AST (U/L)		29.60 (22.10, 37.45)	24.10 (20.00, 32.47)	0.011
TBIL (umol/L)		20.80 (15.00, 30.15)	20.60 (13.30, 27.42)	0.436

*Postoperative*				
RBC		3.36 (0.58)	3.53 (0.67)	0.062
PLT		76.00 (60.50, 97.50)	83.00 (59.00, 100.00)	0.708
RDW1 (%)		15.40 (14.60, 17.05)	16.90 (15.10, 19.65)	0.004
RDW3 (%)		15.40 (14.55, 17.00)	18.65 (14.97, 21.30)	<0.001
RDW7 (%)		15.60 (14.40, 17.20)	16.00 (14.78, 19.20)	0.044
MPV1 (%)		11.30 (10.24, 12.40)	11.05 (10.30, 11.95)	0.818
MPV3 (%)		11.40 (10.07, 12.40)	11.15 (10.40, 12.70)	0.498
MPV7 (%)		10.79 (1.36)	10.94 (1.51)	0.459
PDW1 (%)		17.10 (16.18, 17.85)	17.10 (16.28, 17.92)	0.853
PDW3 (%)		16.40 (15.93, 16.95)	16.58 (15.87, 17.17)	0.443
PDW7 (%)		16.10 (15.38, 16.40)	15.90 (14.40, 16.31)	0.229

Bold indicates *p* value < 0.05. BMI: body mass index, PVD: portal vein diameter, PVV: portal vein velocity, MELD: model for end-stage liver disease, TBIL: total bilirubin, WBCs: white blood cell, RBC: red blood cell, HB: hemoglobin, PT: prothrombin time, PLT: platelet, APTT: activated partial thromboplastin time, FIB: fibrinogen, TT: thrombin time, HCT: hematocrit, MPV: mean platelet volume, PDW: platelet distribution width, RDW: red blood cell distribution, AST: aspartate aminotransferase, and ALT: alanine aminotransferase.Numbers 1, 3, and 7 represent the factors on a postoperative day (POD) 1, 3, and 7, respectively.

**Table 2:** Multivariable logistic regression.

Factors	Coefficients	OR	95%CI	*P* value
Bleeding history	1.226	3.407	1.670–7.220	<0.001
Length of spleen	0.015	1.015	1.001–1.029	0.034
PVD	0.327	1.387	1.203–1.642	<0.001
RDW3	0.172	1.188	1.073–1.326	0.001

OR: odds ratio, CI: confidence interval, PVD: portal vein diameter, and RDW3: red blood cell on POD 3.

**Table 3:** AUC of the risk factors.

Factors	AUC	Cut-off	95%CI	Sensitivity	Specificity
RDW3	0.685	17.95	0.604–0.766	0.592	0.832
PVD	0.757	13.65	0.684–0.829	0.711	0.765
Spleen length	0.655	185.5	0.573–0.737	0.513	0.790
Bleeding history	0.603	0.396	0.532–0.674	0.592	0.613
Combined	0.830	0.44	0.770–0.890	0.711	0.849

AUC: area under the curve, OR: odds ratio, CI: confidence interval, PVD: portal vein diameter, and RDW3: red blood cell on POD 3.

## Data Availability

The datasets used and/or analyzed during the current study are available from the corresponding author upon reasonable request.

## Supplementary Materials

Supplementary Materials Supplementary Table 1. Location of the PVST in 76 patients.
