# Supplementary material for: Nomogram for Predicting Postoperative Portal Venous Systemic Thrombosis in Patients with Cirrhosis Undergoing Splenectomy and Esophagogastric Devascularization
Source: Can J Gastroenterol Hepatol. 2022 Nov 4;2022:8084431. doi: 10.1155/2022/8084431 (PMC9652084; doi:10.1155/2022/8084431)
Supplement: Supplementary Materials — Supplementary Table 1. Location of the PVST in 76 patients. [file 8084431.f1.doc]

**Supplementary Table 1.** Location of the PVST in 76 patients

| Localization | Cases |
| --- | --- |
| Portal Trunk | 34 |
| Left Branch | 8 |
| Right Branch | 6 |
| Splenic Vein | 1 |
| Portal Trunk+Left Branch+Right Branch | 5 |
| Portal Trunk+Right Branch | 9 |
| Portal Trunk+Left Branch | 6 |
| Portal Trunk+ Superior Mesenteric Vein | 1 |
| Portal Trunk+Splenic Vein | 3 |
| Portal Trunk+Right Branch+Splenic Vein | 2 |
| Right Branch+Left Branch | 1 |
| Total | 76 |
